# Conservative Surgery of the Face

**Published:** 1872-11

**Authors:** 


### ARTICLE VI.

### *Conservative Surgery of the Face.*

The subjoined instructive case is reported by Dr. Meldon, in the Dublin *Medical Journal*:—

Thomas Dillon, aged thirty-five years, was conveyed to the hospital on September 3d, 1870. As a laborer he was employed raising heavy weights on board vessels by means of a windlass; whilst so occupied the handle slipped, and flying round struck him on the face with tremendous force. On admission into the hospital he was quite insensible, and the face presented a frightful appearance. He had evidently been struck from the angle of the mouth on the right to the edge of the orbit on the left side. Both upper maxillary bones were fractured. The nasal bones pulverized; the prominent portion of the orbit flattened; the palate comminuted, and all the upper teeth, with their alveolar process, and portions of the upper maxillary bones, hung loosely in the wound. The inferior maxillary bone was also fractured. The large flap containing part of the nose and upper lip were turned down over the chin and neck, suspended by two narrow portions of skin and muscle. A good deal of venous oozing was checked by cold. The upper teeth were replaced, and the soft parts brought as nearly as possible together, and kept in their place by means of sutures, together with an ordinary mask and a bandage. The right eye was found much chemosed, and although the lid was divided it escaped all further injury. When the patient recovered sensibility it was found he could not swallow, but on the following day when the palate was fixed by means of a gutta-percha plate, power of swallowing returned. The patient made a rapid recovery, and left the hospital with a very good face. The teeth and bone attached remained for a considerable time so loose that they could easily have been removed by the finger and thumb; they have now, however, become quite firm, and he has made an excellent recovery. Some months after his leaving the hospital I performed a successful operation for entropion, caused by contraction after the accident; of the entropion there now remains not a trace to be seen.—*Med. and Surg. Reporter.*

---

